# TMVR for the Treatment of Mitral Regurgitation: A State-of-the-Art Review

**DOI:** 10.1161/CIRCINTERVENTIONS.125.015298

**Published:** 2025-09-10

**Authors:** Francesco Tartaglia, Giulia Antonelli, Mauro Gitto, Kamil Stankowski, Dario Donia, Giulio Stefanini, Azeem Latib, Antonio Colombo, Antonio Mangieri, Mauro Chiarito

**Affiliations:** 1 Department of Biomedical Sciences, Humanitas University, Pieve Emanuele-Milan, Italy (F.T., G.A., M.G., K.S., D.D., G.S., M.C.).; 2 Cardio Center, Istituto di Ricovero e Cura a Carattere Scientifico (IRCCS) Humanitas Research Hospital, Rozzano-Milan, Italy (F.T., G.A., M.G., K.S., D.D., G.S., A.C., A.M., M.C.).; 3 Department of Interventional Cardiology, Montefiore Einstein Center for Heart and Vascular Care, Montefiore Medical Center, New York, NY (A.L.).

**Keywords:** heart ventricles, mitral valve, patient selection, risk

## Abstract

Mitral regurgitation is the most common valve disease worldwide. Despite its wide success in inoperable or high-risk surgical patients, transcatheter edge-to-edge repair remains limited by some anatomic features and the non-negligible rate of significant residual regurgitation. Transcatheter mitral valve replacement has emerged as a viable alternative that promises to overcome these issues, but its development has been progressing slowly. This review aims to provide a comprehensive overview of the current state of transcatheter mitral valve replacement, including patient selection, procedural techniques, and currently available outcomes.

Mitral regurgitation (MR) is the most common valvular disease worldwide. Because up to half of the patients are ineligible for surgery due to high operative risk,^1^ transcatheter edge-to-edge repair (TEER) has become a validated option for high-risk patients, and is currently the first-line percutaneous therapy for degenerative (recommendation class IIb and IIa in the European and American guidelines, respectively)^2,3^ and functional severe symptomatic MR (class IIa according to both guidelines; Figure 1).

**Figure 1.**
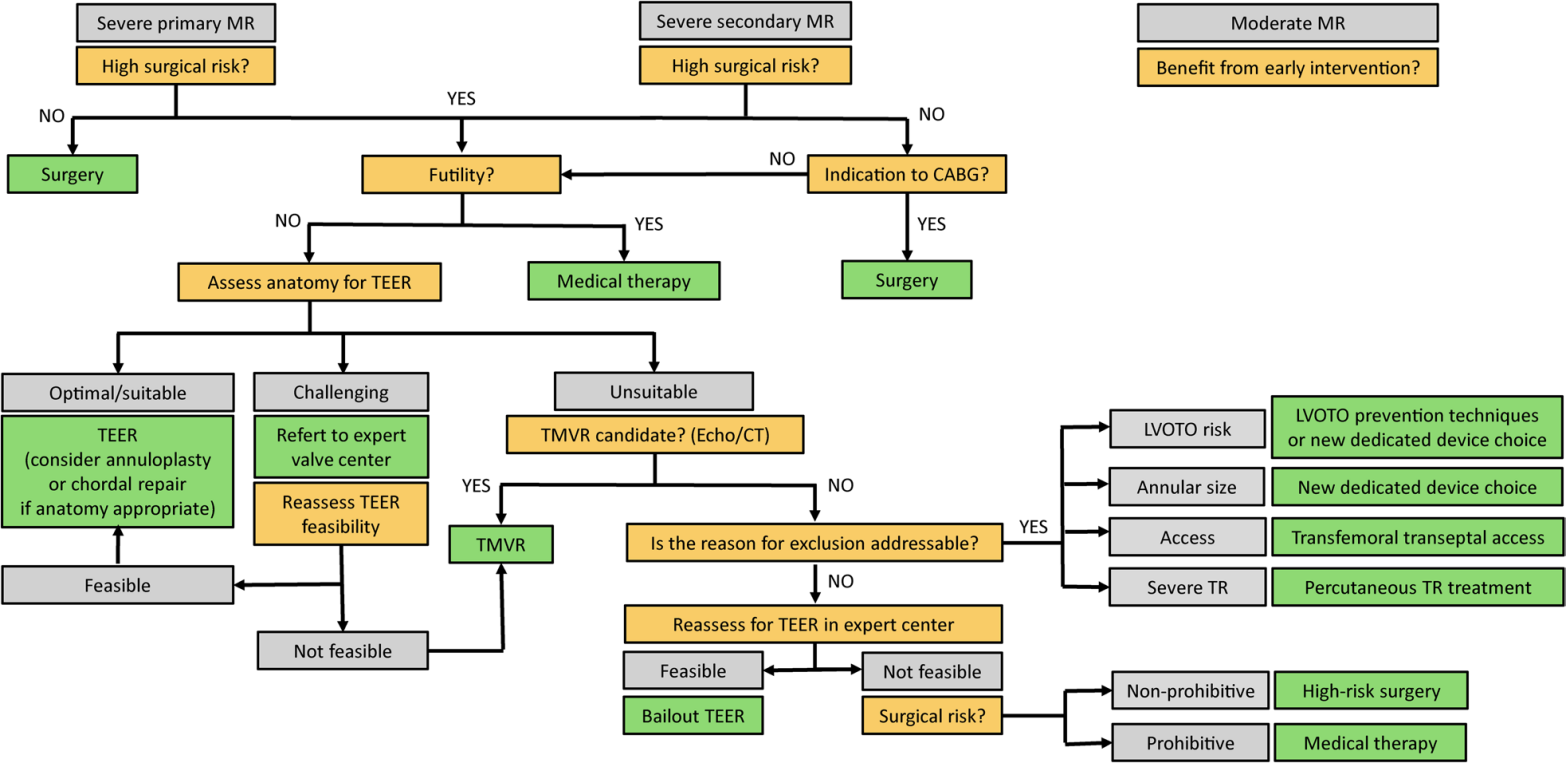
**Treatment of mitral regurgitation (MR): a simple algorithm.** The algorithm shows when to consider transcatheter mitral valve replacement (TMVR). CABG indicates, coronary artery bypass grafting; CT, computed tomography; echo, echocardiography; LVOTO, left ventricular outflow tract obstruction; TEER, transcatheter edge-to-edge repair; and TR, tricuspid regurgitation.

However, the success of TEER largely depends on mitral valve (MV) anatomy.^4^ Hence, inadequate MR reduction is frequent in the case of complex MV disease (up to 37% of ≥ moderate MR at 1 year), limiting the symptomatic improvement and prognostic benefit of TEER.^5,6^

Transcatheter MV replacement (TMVR) has the potential to overcome these limitations, but is hampered by several technical challenges. We present an overview of TMVR features and devices, offering a perspective on future technological developments.

## TMVR for MR: A Luxury for the Few?

**Figure 2.**
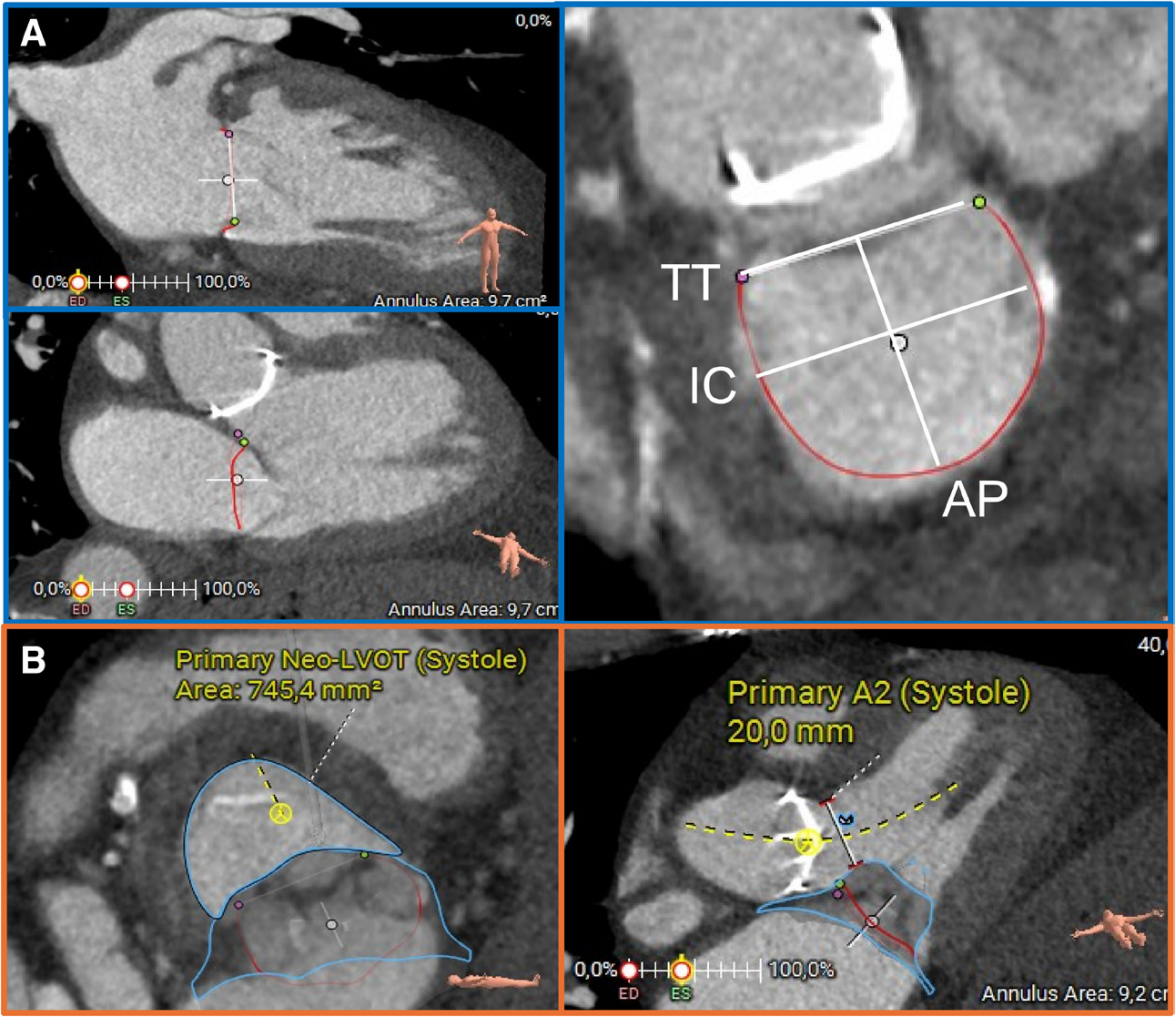
**Cardiac computed tomography showing measurement of mitral annulus and neo–left ventricular outflow tract (LVOT).** Screening for Tendyne in a patient with previous surgical aortic valve replacement and myectomy. **A**, D-shaped annulus with relevant measurements in 2-chamber view (**upper left**), 3-chamber view (**lower left**), and short axis (**right**): trigone-to-trigone (TT) distance, intercommissural (IC) distance, and antero-posterior (AP) distance. **B**, Neo-LVOT area (**left**) and diameter (**right**).

TMVR devices have been developed for the treatment of anatomically complex Mr Multiple devices have been designed, but their clinical implementation has been slow, primarily because of the need for transapical access. Moreover, TMVR is currently limited by a high screening failure rate, with exclusion being reported in up to 70% of candidates and mainly linked to anatomic limitations (eg, annular size, left ventricle [LV] size, risk of LV outflow track obstruction [LVOTO], mitral annulus calcification [MAC], and leaflet morphology) as assessed through echocardiography and cardiac computed tomography (Figure 2).^5,6^

When set against TEER, both procedures have advantages and disadvantages (Table 1). As for clinical outcomes, a propensity-matched retrospective analysis of 2 large European registries showed comparable outcomes between transfemoral TMVR and TEER in complex anatomies.^7^ Functional capacity was significantly better in the TMVR group, but no benefit on mortality (25.8% in the TMVR group versus 18.9% in the TEER group) or heart failure hospitalizations (24.6% versus 19.7%) was observed. Two-year outcomes from a small cohort of patients have been recently presented^8^: 30-day mortality was increased with TMVR, mostly due to periprocedural complications, while the survival curves tended to overlap at 2 years.

**Table 1.**
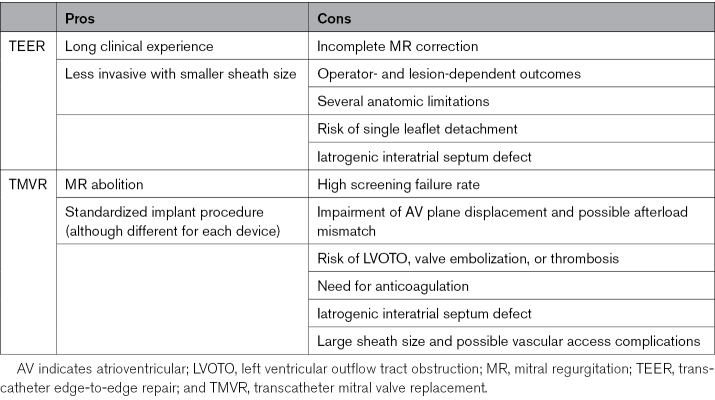
TMVR Versus TEER: Pros and Cons

The ongoing SUMMIT (Clinical Trial to Evaluate the Safety and Effectiveness of Using the Tendyne Mitral Valve System for the Treatment of Symptomatic Mitral Regurgitation; URL: https://www.clinicaltrials.gov; Unique identifier: NCT03433274) trial is the first randomized study comparing TMVR with the Tendyne device (Abbott, Menlo Park, CA) against TEER with the MitraClip device (same manufacturer) in patients with severe or moderate-to-severe MR.

## TMVR Challenges

Table 2 summarizes the developments in addressing TMVR procedural challenges. These represent the gatekeeper limiting TMVR to a complex second-line treatment that few patients can benefit from and currently preventing it from becoming a widely adopted solution.

**Table 2.**
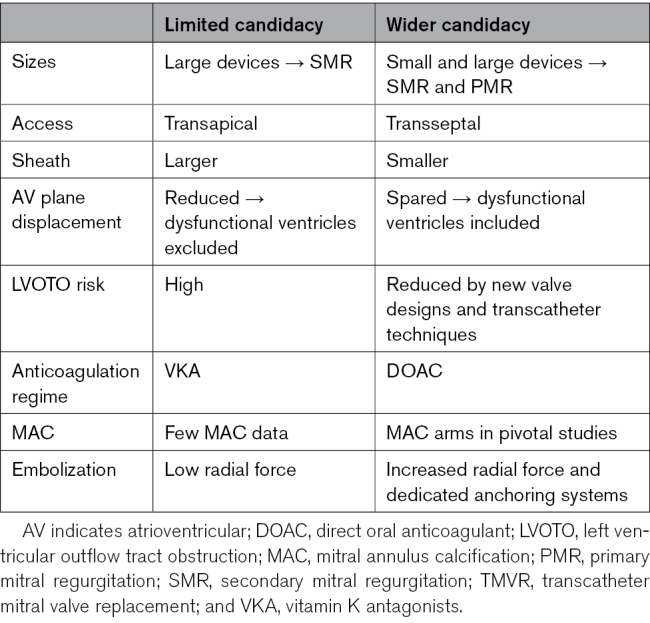
Improvements That Might Widen Candidacy to TMVR

### Access and Atrial Septal Defect

In spite of the risk of vascular complications, the transfemoral approach is associated with a reduced rate of complications and increased survival compared with the transapical route.^9^ Hence, most manufacturers are converting their transapical devices toward the transseptal approach, at the expense of larger external sheath diameters if compared with TEER (32F or 33F versus 22F or 24F).^10^ The residual atrial septal defect is usually closed in case of bidirectional or right-to-left shunts and atrial septal defects >8 mm.^11^

### Anticoagulation Regimen

Valve thrombosis is reported in 9% of TMVR cases.^12^ Three months of anticoagulation with vitamin K antagonists are currently recommended after TMVR.^2,3^ However, in a small single-center observational study, the use of direct oral anticoagulants after TMVR was associated with reduced major bleeding events (9%, versus 35% in the vitamin K antagonist group) and hospitalization length, with similar incidence of valve thrombosis.^13^

## Left Ventricular Outflow Tract Obstruction

### Assessing the Risk of LVOTO

LVOTO is a feared complication occurring in ≈7% to 9% of TMVR procedures, defined as an increase in mean left ventricular outflow tract (LVOT) gradient of ≥10 mm Hg from baseline or a peak LVOT gradient >30 mm Hg (hemodynamically significant if >50 mm Hg) as assessed by echocardiography.^14^

For optimal LVOTO risk stratification, it is essential to evaluate aorto-mitral annular angle, LV geometry, septal hypertrophy, anterior mitral leaflet (AML) length, and the geometry of prosthesis, as well as other device-specific measures.^15,16^

The transcatheter heart valve (THV) creates a neo-LVOT, defined by the anteriorly displaced AML, the THV stent, and the basal to mid anteroseptal left ventricular wall. Neo-LVOT area can be reconstructed by cardiac computed tomography on patient- and device-specific basis (Figure 2). In case of skirted prostheses, the neo-LVOT should be measured at the level of the skirt (skirt neo-LVOT), as it can contribute to the risk of LVOTO.^15^ A neo-LVOT area ≤1.7 cm² has been shown to predict LVOTO, but this cutoff is still matter of debate.^15,17^ Measurements are made in the mid- to end-systolic phase, when LVOT is at its smallest size. However, the neo-LVOT is larger in early systole. Ongoing studies are assessing the most accurate phase for neo-LVOT measurement, because considering the early systolic neo-LVOT may be more reliable and increase the eligibility of patients for TMVR.^18^

The aorto-mitral annular angle significantly impacts the extension of the prosthesis into the LVOT. Angles close to 90° increase the risk of LVOTO, as well as septal hypertrophy (>15 mm) and small left ventricular end-diastolic diameter (<48 mm).^17^ Lastly, long AML (>25 mm) may cause dynamic LVOTO due to systolic anterior motion.^17^

### Prevention of LVOTO

**Figure 3.**
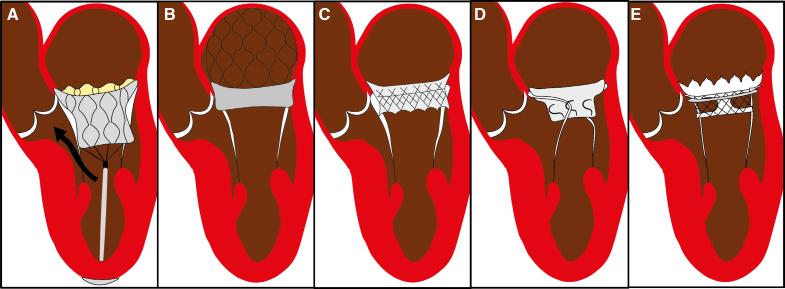
**Schematic representation of main valve design features to prevent left ventricular outflow tract obstruction (LVOTO).** High-profile valves (such as Tendyne [Abbott]) have an increased risk of LVOTO, as blood is forced to pass through the limited space between valve and septum (black arrow; **A**). The main mechanisms designed to prevent LVOTO include: supra-annular design, as in AltaValve (4C Medical Technologies), which minimizes ventricular protrusion by anchoring to the left atrium (**B**); a short ventricular profile, as in Intrepid (Medtronic; **C**); displacement or grasping of anterior mitral leaflet, as in Innovalve (Edwards Lifesciences; **D**); a modified skirt, as in HighLife Clarity (HighLife SAS; **E**).

Different valve design features have been developed to prevent LVOTO (Figure 3). If the risk persists beyond valve choice, several percutaneous strategies have been suggested to avoid LVOTO (Table 3). Some patients may report symptomatic improvement after these procedures, even before valve replacement.

**Table 3.**
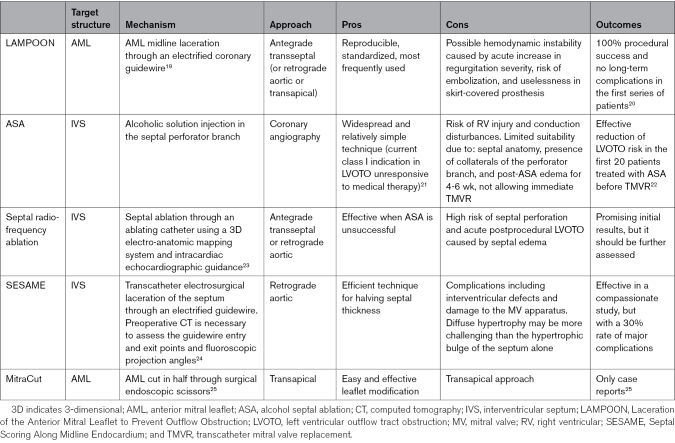
LVOTO Prevention Techniques

## TMVR Devices

**Figure 4.**
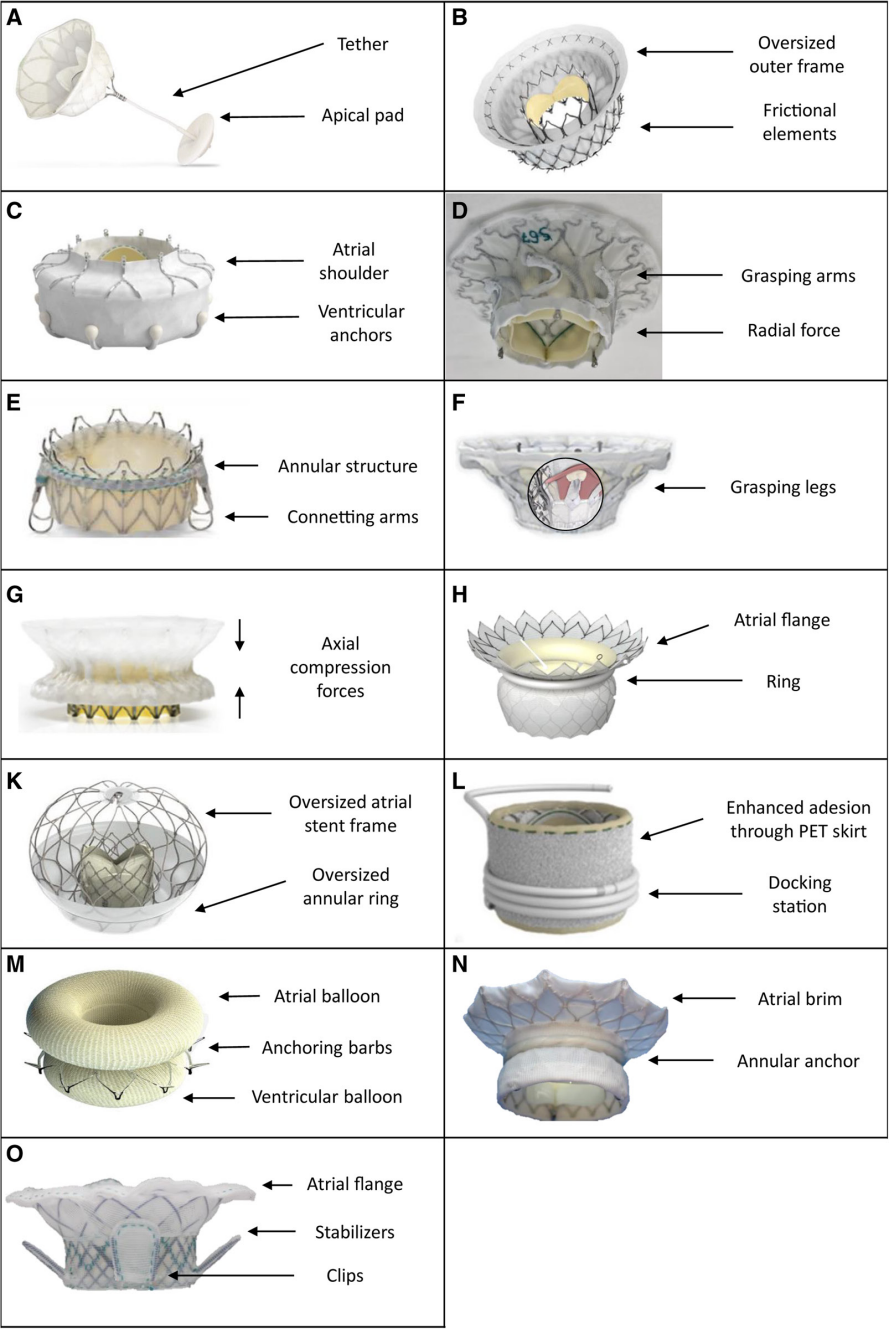
**Current transcatheter mitral valve replacement devices and main anchoring mechanisms.** (**A**) Tendyne. (**B**) Intrepid. (**C**) EVOQUE Eos. (**D**) Innovalve. (**E**) Saturn. (**F**) Cardiovalve. The circle shows inner composition of the valve to demonstrate leaflet grasping by ventricular legs. (**G**) Cephea. (**H**) HighLife. (**I**) AltaValve. (**L**) SAPIEN M3. (**M**) ValvSync. (**N**) Tioga. (**O**) Palmetto valve. PET indicates polyethylene terephthalate.

In contrast with the limited variety of TEER devices, each TMVR prosthesis presents specific anchoring mechanism and deployment procedure. Here, we report a list of the most widely used and currently available TMVR devices (Figure 4). For each valve, specific characteristics are summarized in Table 4, and results of available clinical studies are outlined in Table 5.

**Table 4.**
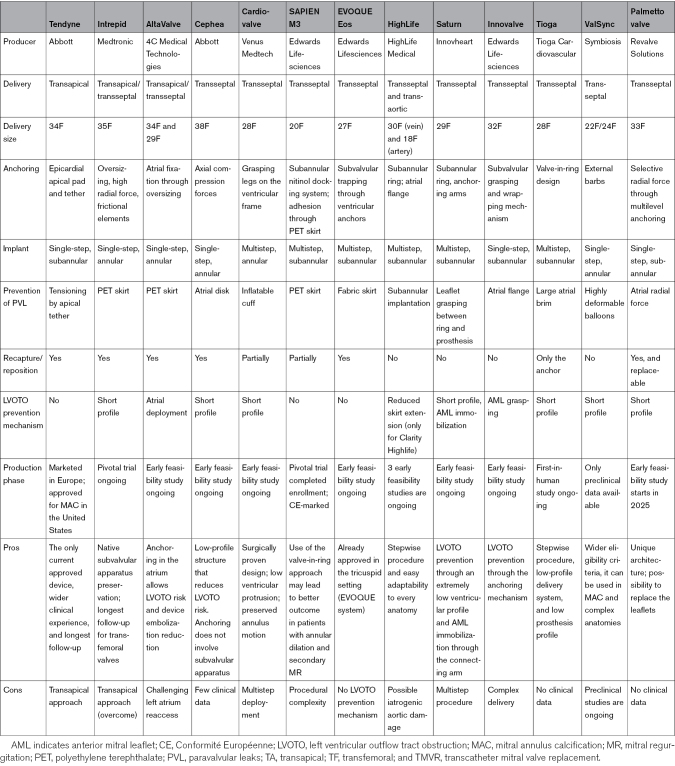
TMVR Devices

**Table 5.**
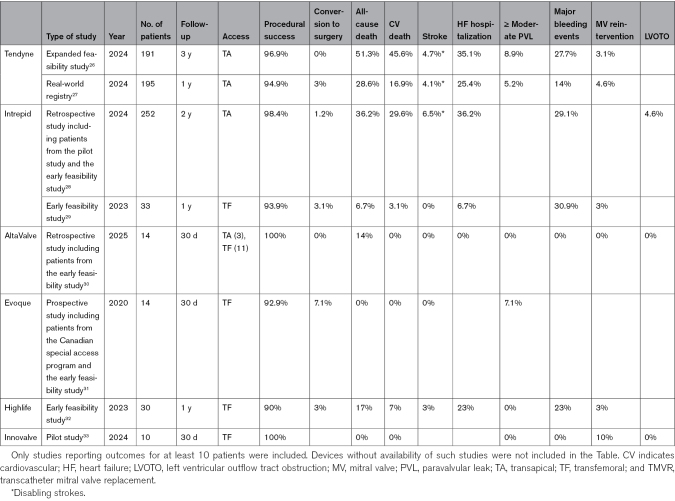
Results of Available Clinical Study for Each TMVR Device

### Tendyne

The Tendyne device (Abbott, Menlo Park, CA) is a self-expanding, completely retrievable and repositionable THV, composed of 3 porcine pericardial leaflets attached to a self-expanding nitinol bioprosthesis. The inner frame holds the valve, whereas the flexible outer frame (septo-lateral diameter: 29–40 mm; intercommissural diameter: 42–52 mm) forms a D-shape to fit the mitral annulus, with a raised cuff to align with the mitro-aortic continuity and prevent paravalvular leak (PVL). The valve is implanted transapically and secured by a high-molecular-weight polyethylene tether passing through the left ventricular apex and anchored to an epicardial pad, without need for rapid pacing during deployment.^34^

The first Global Feasibility Study conducted in 100 patients had encouraging rates of technical success (>97%), MR reduction (93.2% of patients with none/trivial MR at 1 year) and clinical outcomes (2-year cardiovascular mortality and heart failure–hospitalization rate: 34% and 38.8%, respectively). Device-related adverse events occurred in around 20% of patients and mainly in the first year after the procedure, whereas 9 patients experienced PVL during the first year post-TMVR, and 7 of them were treated conservatively.^12^

The Expanded Clinical Study (https://www.clinicaltrials.gov; Unique identifier: NCT02321514) included 91 additional patients. At 3 years, cardiovascular mortality was 45.6% and major bleeding occurred in 27.7% of patients. Incidence of PVL was 8.9%, with 5 cases needing reintervention. A significant improvement in functional status was also reported.^26^ Institutional experience with the device was found to be an independent predictor of early mortality.^35^

Real-world studies confirmed the feasibility of TMVR with Tendyne. In 1 center, among 174 patients screened for eligibility, 63 patients were approved, and only 17 eventually underwent Tendyne implantation, with suboptimal clinical results (94% technical success rate, 17.7% in-hospital mortality, and 35.3% 1-year mortality).^36^

The TENDER (Tendyne European Experience) is an ongoing multicenter real-world registry enrolling patients undergoing TMVR with Tendyne. Screening failure rate was as high as 55%, with high risk of LVOTO accounting for half of the cases. Technical success was high with a consistent and stable reduction of MR degree at 1 year: 97.6% of the 189 treated patients had mild or less MR after Tendyne TMVR. All-cause mortality and cardiovascular mortality at 1 year occurred in 28.6% and 16.7% of patients, respectively, with a 25.4% incidence of heart failure hospitalizations.^27^

As described above, the SUMMIT trial is comparing TMVR with Tendyne against TEER with MitraClip.

To date, Tendyne is the first and only TMVR device approved for commercial use in Europe, but only for valve-in-MAC in the United States. Its leadership in the field of TMVR could be questioned because its anchoring mechanism is strictly linked to the transapical access.

### Intrepid

The Intrepid system (Medtronic, Minneapolis, MN) includes a bioprosthesis and a transapical 35F delivery system. The bioprosthesis features a trileaflet bovine pericardial valve (27 mm) within a self-expanding nitinol frame with a dual-structure design. The circular inner stent houses the valve, while the outer fixation stent (42 or 48 mm; 54 mm under development) engages the mitral annulus. The outer frame is flexible in its atrial portion (to conform to the native annulus) and relatively stiff in the ventricular portion (to resist against compression). Fixation is obtained by a mix of different mechanisms: increased radial force through valve oversizing (10–30%) and friction exerted by the rings of small cleats on the outer frame. The design preserves the native leaflets and chordae, using them to obtain a sealing effect around the device. A polyester fabric skirt on both stent frames helps prevent PVL and promotes tissue ingrowth for long-term fixation. Risk of LVOTO is reduced by a short profile (<18 mm and further reduced in the second generation). Valve release requires rapid ventricular pacing.^37^

In the first pilot trial (50 patients), the 30-day mortality rate was 14%, with a procedural success rate of 98% and mild or no residual MR achieved in all patients.^38^

The APOLLO trial (Transcatheter Mitral Valve Replacement With the Medtronic Intrepid TMVR System in Patients With Severe Symptomatic Mitral Regurgitation; URL: https://www.clinicaltrials.gov; Unique identifier: NCT03242642) is an ongoing pivotal, single-arm open-label trial that investigates the use of Intrepid TMVR in high surgical risk patients with complex MV disease deemed unsuitable for TEER.

A recent retrospective analysis of the transapical TMVR system on 252 patients from both the pilot study and the APOLLO trial showed a 98.4% success rate in device implantation.^28^ Notably, although CT-derived neo-LVOT was not indicative of high LVOTO risk, 11 patients experienced LVOTO, and 3 of them died. Mortality rate at 30 days was 13.3%, with at least 1 death out of 4 related to transapical access complications. At 2 years, all-cause mortality and heart failure hospitalization were both reported in 36.2%. As for ischemic and bleeding valve-related events, stroke incidence was 9.3%, clinically relevant thrombosis was reported in 3% and major bleeding events occurred in 29% of patients.

To reduce the morbidity associated with the transapical approach, a 35F transfemoral transseptal TMVR delivery system was developed, later reduced to 29F. In the feasibility study conducted on 33 patients treated via the 35F transfemoral transseptal Intrepid TMVR, procedural success was 93.9%, with 96% of patients presenting no MR at 1 year. There were 9 MVARC (Mitral Valve Academic Research Consortium)-defined bleeding events, 8 of which were access-related. The 30-day and 1-year all-cause mortality rates were low (0% and 6.7%, respectively),^20^ whereas 22.3% of patients were hospitalized for cardiovascular reasons at 1 year.^29^

These results make the Intrepid valve the transfemoral transseptal TMVR system with the longest follow-up data currently available, but longer-term data are needed.

### AltaValve

**Figure 5.**
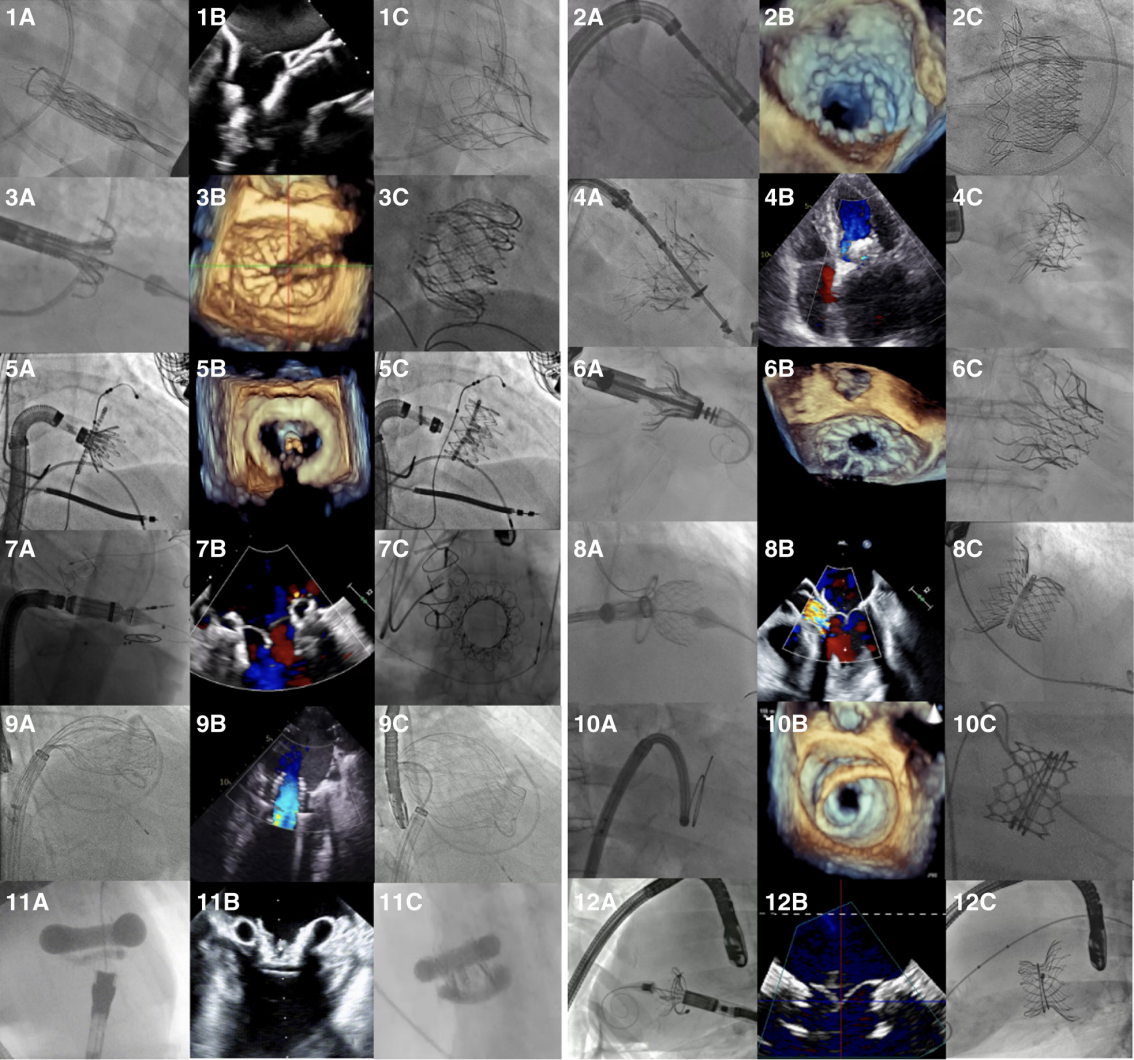
**Implantation of currently available devices.** For each valve, image **A** shows intraprocedural angiography, image **B** shows intraprocedural or postprocedural echocardiography, and image **C** shows the device released at the end of the procedure. (1) Tendyne; (2) Intrepid; (3) EVOQUE; (4) Innovalve; (5) Saturn (courtesy of InnovHeart); (6) Cardiovalve; (7) Cephea; (8) HighLife (courtesy of HighLife Medical); (9) AltaValve; (10) SAPIEN M3; (11) ValSync (animal model; courtesy of Symbiosis); (12) Tioga (animal model).

The AltaValve (4C Medical Technologies, Minneapolis, MN) is a spherical, supra-annular TMVR device that passively fixates in the left atrium (Figure 5, 1A–1C). It was developed with the aim of minimizing device embolization and LVOTO risk, because it has minimal ventricular protrusion. The THV comprises a self-expanding nitinol stent frame that conforms to left atrium anatomy, and a 27-mm trileaflet bovine pericardial valve placed inside. The annular ring is located at the ventricular end of the THV, and it is covered by a fabric skirt that ensures valve sealing. The ring is oversized compared with the MV annulus to facilitate anchoring and is available in 3 different dimensions (40, 46, and 54 mm).^39^ The stent frame has wide cells with the aim to allow future transseptal access to the left atrium, although it will be more complex to perform. The delivery is possible via both transapical (34F) and transfemoral (29F) access.

Recent results from a small cohort of 14 patients (11 transseptal, 3 transapical) showed 100% technical success and MR reduction to none or mild in all cases. Only 2 patients required atrial septal defect closure. Two nonvalve-related deaths were reported at 30 days, without any stroke or reinterventions.^30^

The early feasibility study investigating the performance and safety of AltaValve device for severe MR treatment is ongoing, and the pivotal trial will begin soon.^40^

Caging the left atrium will impede its contractile function, but most of these patients already have permanent atrial fibrillation before implant. A possible preventive effect on atrial enlargement can be speculated.

### Cephea

The Cephea (Abbott, Menlo Park, CA) is a TMVR device with transseptal implantation, composed of a self-expanding bioprosthesis contained in a nitinol double-disc frame (Figure 5, 2A–2C). The outer ring conforms to mitral annular anatomy, whereas the inner ring isolates and contains the trileaflet bovine pericardial valve. Anchoring is based only on the axial compression of the mitral annulus from the ventricular and the atrial components of the valve. Three different sizes (32, 36, and 40 mm) are currently available. The low ventricular protrusion helps prevent LVOTO.

Clinical experience is limited to a few reported cases, but the feasibility trial is ongoing (URL: https://www.clinicaltrials.gov; Unique identifier: NCT05061004).^41,42^

More data are needed to evaluate if the unique anchoring mechanism and the low valve profile will translate into a clinical benefit.

### CardioValve

The CardioValve (Venus Medtech, or Yehuda, Israel) is a 3-step deployment self-expanding TMVR device implanted through a transseptal route. The THV consists of a trileaflet bovine pericardial bioprosthesis included in an atrial and a ventricular frame. Both frames have 12 grasping legs. The ventricular frame grasps the ventricular facet of the mitral leaflets (step 1), then the leaflets are captured by delivery the atrial flange (step 2), and the inner bioprosthesis is finally deployed (step 3). Prevention of PVL is achieved through an inflatable cuff solution: the blood from the ventricle inflates the atrial flange, which is thus sealed toward the annulus.

The valve is implanted through a 28F sheath and its low ventricle protrusion design reduces LVOTO risk (12 mm of ventricular protrusion in a 32 mm-tall valve). Three different sizes are available to fit most anatomies (medium, large, and extra-large, which cover up to 55 mm). A new delivery system has been successfully tested in the first-in-human experience.

Current American (AHEAD US [Cardiovalve Transfemoral Mitral Valve System] URL: https://www.clinicaltrials.gov; Unique identifier: NCT03813524) and European (AHEAD European [European Feasibility Study of the Cardiovalve Transfemoral Mitral Valve System]; URL: https://www.clinicaltrials.gov; Unique identifier: NCT03339115) feasibility trials are ongoing. Current evidence is limited to a small case series.^43,44^

The main strengths of this THV are the anchoring mechanism, which does not rely on radial forces and could thus allow annulus remodeling and preserve annulus motion, and the possibility of covering a wide range of annular sizes. Moreover, the low profile provides little interference with the LVOT.

### SAPIEN M3

The SAPIEN M3 (Edwards Lifesciences, Irvine, CA) is a 2-component TMVR system including a modified SAPIEN 3 valve and a docking system (Figure 5, 3A–3C). The SAPIEN M3 valve is a 29 mm balloon-expandable bovine pericardial valve, different from the SAPIEN 3 aortic valve, presenting a knitted polyethylene terephthalate skirt which assists in anchoring (by increasing friction with the dock station) and leak prevention. The nitinol dock is composed of 1 ventricular turn (that guides the encircling of native leaflets), 1 atrial turn (that maintains dock position), and several functional turns in between (that provide the anchor for the THV). The procedure consists of 2 steps: first, the nitinol dock encircles the chordae tendineae below the mitral annular plane to support the valve deployment; then, the THV is implanted transseptally through the same 20F sheath.

Only the first-in-human case has been published, but the pivotal trial (SAPIEN M3 system ENCIRCLE trial [Transcatheter Mitral Valve Replacement via Transseptal Access]; URL: https://www.clinicaltrials.gov; Unique identifier: NCT04153292), which also includes an arm of patients with previously failed TEER and an arm enrolling patients with MAC, has completed enrollment.^45,46^ Previous studies using aortic balloon-expandable SAPIEN S3 showed the potential of these valves for treating MAC, but specific data on SAPIEN M3 in this context are lacking.^47^ The valve has recently received the Conformité Européenne mark.

The docking station transforms the procedure into a valve-in-ring–like procedure. This may lead to better outcomes in patients with secondary MR and annular dilation, at the expense of increased procedural complexity.

### EVOQUE Eos

The EVOQUE Eos system (Edward Lifesciences, Irvine, CA) is a self-expanding THV deployed transseptally through a 27F sheath (Figure 5, 4A–4C). The bioprosthesis consists of a trileaflet bovine pericardial valve, a self-expanding nitinol frame with an atrial shoulder and an intra-annular sealing skirt, and 2 different valvular sizes are currently available (44 and 48 mm). It derives from a redesign of the EVOQUE valve (approved for commercial use in the United State as a tricuspid valve replacement device) and is fully retrievable and recapturable.

The ventricular component is released first, followed by the atrial shoulders in a multistep implant. The fixation mechanism includes several anchors incorporated in the nitinol frame that trap the leaflets and the chordae tendineae against the nitinol frame. The shoulders provides fixation in the atrial portion, and the skirt reduces PVL.

Early outcomes of 14 patients treated with the EVOQUE system (before the development of the EVOQUE Eos) revealed a technical success rate of 92.9%. One patient experienced severe PVL requiring surgical conversion, and 3 patients experienced mild or moderate PVL, requiring percutaneous closure in 2 cases. No residual MR was registered in 83% of patients. Eleven patients underwent atrial septal defect closure. One disabling stroke and no death were registered at 30 days.^31^

The MISCEND (Edwards EVOQUE Eos Mitral Valve Replacement: Investigation of Safety and Performance After Mitral Valve Replacement With Transcatheter Device; URL: https://www.clinicaltrials.gov; Unique identifier: NCT02718001) early feasibility study is ongoing and aims to investigate efficacy of the EVOQUE Eos system. Early reports on 60 patients showed reduction of MR to mild or less in all patients at 1 year.^48^

### HighLife

The HighLife (HighLife SAS, Paris, France) is a 2-component TMVR system consisting of a self-expanding bioprosthesis and a subvalvular ring. The ring is available in 1 size and is deployed through the transaortic retrograde route. It captures the native leaflets and participates in anchoring for the THV, avoiding slipping and valve embolization. The THV is a 28 mm trileaflet bovine pericardial bioprosthesis implanted through a transvenous transseptal approach after the ring has been deployed. The largest version of the valve can be implanted in native annuli up to 53 mm in diameter.^49^ In the second iteration of the valve (HighLife Clarity), some portions of the skirt have been removed to reduce the risk of LVOTO, the most common cause of screening failure in early reports.^50^

The prospective feasibility study on 30 patients reported a 90% procedural success rate, with only 1 patient requiring conversion to open-heart surgery due to valve embolization. At 1 year, 13% of patients necessitated atrial septal defect closure. The survival rate was 90% at 30 days and 83% at 1-year follow-up. Ultimately, 78% of patients who underwent successful implantation of the HighLife device had no or minimal MR, and 22% exhibited mild MR at 1 year.^32^ Three feasibility trials (https://www.clinicaltrials.gov; Unique identifiers: NCT04029337 and NCT04029363 for Highlife in the United States and Europe, respectively; URL: https://www.clinicaltrials.gov; Unique identifier: NCT04888247 for HighLife Clarity) are currently ongoing, while the US pivotal trial will begin in 2025. The Food and Drug Administration recently granted the Breakthrough Device Designation for this device.

The stability offered by the valve-in-ring-like anchoring is the main strength of this valve, but the implant procedure needs an additional arterial access.

### Saturn

The Saturn (Innovheart, Milan, Italy/ Boston, MA) is a novel TMVR system. The bioprosthesis consists of an annular structure and a central valve with 2 connecting arms. It is available in 2 different dimensions (28 and 31 mm) and is designed to be deployed via a transapical access or via a percutaneous transseptal 29F approach through a multistep procedure.^51^ The annular structure is positioned behind the native mitral leaflets, reshaping the annulus and providing anchoring for the 2 connecting arms of the central valve.

The Saturn system was designed to have an extremely low ventricular profile (13 mm) aimed at overcoming TMVR screening failure due to LVOTO risk. Additionally, the annulus ring and the anterior connecting arm prevent dynamic obstruction due to systolic anterior motion, immobilizing the AML.

Limited evidence on the Saturn system is available^52^ and the feasibility trial (CASSINI-EU study [Reduction of Mitral Regurgitation With the SATURN Trans-Septal Transcatheter Mitral Valve Replacement System in Patients With Severe Symptomatic Mitral Regurgitation]; URL: https://www.clinicaltrials.gov; Unique identifier: NCT06414265) is ongoing.

This valve looks promising for cases with high LVOTO risk because of its double prevention mechanism.

### Innovalve

The Innovalve (Edward Lifesciences, Irvine, CA) is a TMVR device delivered transseptally through a 32F system (Figure 5, 5A–5C). The THV consists of an atrial flange and a self-expanding cylinder that houses a trileaflet bovine pericardium prosthesis with 6 arms. Its anchoring system consists of the arms grasping the subvalvular apparatus and wrapping it with a rotational motion. This adds up to the radial force exerted by the cylinder and the axial force of the atrial flange. The Innovalve design allows LVOTO risk prevention by the grasp of the AML, thus reducing dynamic outflow tract obstruction. The atrial flange covers up to 58.5 mm.

The pilot study on 10 patients (TWIST-GE [TVMR With the Innovalve System Trial—Pilot Study in Georgia]; URL: https://www.clinicaltrials.gov; Unique identifier: NCT05682066) reported a technical success of 100% without in-hospital and 30-day mortality event registered. The global population experienced symptom improvement after the procedure, and a trend toward left systolic function amelioration was reported. However, at 30 days, 9 patients experienced mild or less PVL without laboratory-based hemolysis.^33^

An early feasibility trial (TWIST-EFS [TMVR With the Innovalve System Trial-Early Feasibility Study]; URL: https://www.clinicaltrials.gov; Unique identifier: NCT04919980) is ongoing, and a pivotal trial will soon begin.

The Innovalve TMVR device has a complex delivery, but the rotation maneuver creates tension between the papillary muscle and the mitral annulus, which may favor reverse LV remodeling.^53^

### Tioga

The Tioga Luna valve (Tioga Cardiovascular, Los Gatos, CA) is composed of a fully repositionable and retrievable polymer-covered nitinol anchor that encircles the subvalvular apparatus, and a self-expanding nitinol valve with a large atrial brim (Figure 5, 6A–6C). The 2 components are deployed through a 2-step transseptal procedure using 2 different 28-Fr delivery systems. Moreover, the large atrial brim is intended to reduce PVLs, and the valve profile is low to avoid LVOTO.

After promising preclinical studies, the first-in-human study is currently ongoing.^54^

The valve-in ring design with a stretchable anchor lets the device adapt to different anatomies, but further clinical data are needed.

### ValSync

The ValSync valve (Symbios, Petach Tikva, Israel) is a novel device deployed through a transseptal delivery system in a 3-step procedure (Figure 5, 7A–7C). The system is composed of a bioprosthesis housed into an upper and a lower balloon. The 2 balloons are made of a highly deformable material, and they can be inflated in real time during the procedure to be fully adapted to the surrounding anatomy, up to an annular size of 62 mm. Thus, they create a sandwich effect on the mitral leaflet to anchor the valve. Moreover, they are separated by external barbs that allow the device to anchor to the subvalvular apparatus. Delivery will be done through a low-profile venous sheath (22F–24F).^55^

Only preclinical data are currently available,^56^ but the first-in-human study will begin in 2026.

The innovative mechanism of ValSync allows PVL prevention and aims to allow a wider candidacy, including patients with MAC and other challenging anatomies. The low-profile sheath is another pro of this device.

### Palmetto Valve

The Palmetto Valve (Revalve Solutions Inc, Irvine, CA) is a transseptal TMVR device composed of 2 devices: an adapter that conforms to the mitral annulus, and a minimal leaflet structure that hosts the 3 bovine pericardial leaflets of the THV and is anchored inside the adapter. The internal part can be removed after the implant and replaced with a new one. Both parts are made with a braided wire technology that creates a helical architecture. This should allow the implant to contract and rotate with the heart, encouraging a posteriorly directed blood flow and preserving LV function.^57^

It has a 4-point anchoring mechanism: 2 clips to capture the native leaflets; 2 stabilizers for medial-lateral stability; the atrial flange to seal the annulus; and the valve body to seal the native leaflets. It is implanted through a 33F femoral access and has a low ventricular profile.

First-in-human implant was performed in 2021, and the early feasibility study will begin in 2025. Despite the lack of clinical data, this valve has a novel design and is the first to take into account lifetime management for younger patients.

## Conclusions and Future Directions

TMVR is emerging as an appealing procedure for the treatment of primary and secondary MR, potentially allowing for a sustained reduction in MR severity, but the future of this field remains uncertain, as slow enrollment in clinical studies due to high screening failure has limited the availability of clinical data. Technological improvements of THVs and delivery systems will be the keystone for addressing the problems of patient candidacy and outcomes. Risk of LVOTO is going to be minimized by the diverse prevention mechanisms, and a wide spectrum of annular sizes will be introduced. Latest devices promise to avoid PVL by real-time adaptation to the mitral annulus and to allow simple valve-in-valve procedures in the future, thus allowing the candidacy of low-risk patients. This, in addition to the decrease in complications, which is expected with the reduction in femoral sheath sizes, will be crucial for advancing TMVR from its current niche applicability to a competitive alternative to TEER and surgery.

## ARTICLE INFORMATION

### Acknowledgments

The publication fee for this work was covered by the Italian Ministry of Health’s Ricerca Corrente funding to the IRCCS Humanitas Research Hospital.

### Sources of Funding

None.

### Disclosures

None.
